# Cardiac Electrophysiological Effects of Light-Activated Chloride Channels

**DOI:** 10.3389/fphys.2018.01806

**Published:** 2018-12-17

**Authors:** Ramona A. Kopton, Jonathan S. Baillie, Sara A. Rafferty, Robin Moss, Callum M. Zgierski-Johnston, Sergey V. Prykhozhij, Matthew R. Stoyek, Frank M. Smith, Peter Kohl, T. Alexander Quinn, Franziska Schneider-Warme

**Affiliations:** ^1^Institute for Experimental Cardiovascular Medicine, University Heart Centre Freiburg–Bad Krozingen Medical Center—University of Freiburg, Freiburg, Germany; ^2^Faculty of Medicine University of Freiburg, Freiburg, Germany; ^3^Faculty of Biology University of Freiburg, Freiburg, Germany; ^4^Department of Physiology and Biophysics, Dalhousie University Halifax, NS, Canada; ^5^Department of Pediatrics, Dalhousie University Halifax, NS, Canada; ^6^Department of Medical Neuroscience, Dalhousie University Halifax, NS, Canada; ^7^School of Biomedical Engineering, Dalhousie University Halifax, NS, Canada

**Keywords:** heart, optogenetics, action potential, GtACR1, natural anion channelrhodopsin, zebrafish

## Abstract

During the last decade, optogenetics has emerged as a paradigm-shifting technique to monitor and steer the behavior of specific cell types in excitable tissues, including the heart. Activation of cation-conducting channelrhodopsins (ChR) leads to membrane depolarization, allowing one to effectively trigger action potentials (AP) in cardiomyocytes. In contrast, the quest for optogenetic tools for hyperpolarization-induced inhibition of AP generation has remained challenging. The green-light activated ChR from *Guillardia theta* (GtACR1) mediates Cl^−^-driven photocurrents that have been shown to silence AP generation in different types of neurons. It has been suggested, therefore, to be a suitable tool for inhibition of cardiomyocyte activity. Using single-cell electrophysiological recordings and contraction tracking, as well as intracellular microelectrode recordings and *in vivo* optical recordings of whole hearts, we find that GtACR1 activation by prolonged illumination arrests cardiac cells in a depolarized state, thus inhibiting re-excitation. In line with this, GtACR1 activation by transient light pulses elicits AP in rabbit isolated cardiomyocytes and in spontaneously beating intact hearts of zebrafish. Our results show that GtACR1 inhibition of AP generation is caused by cell depolarization. While this does not address the need for optogenetic silencing through physiological means (i.e., hyperpolarization), GtACR1 is a potentially attractive tool for activating cardiomyocytes by transient light-induced depolarization.

## Introduction

Understanding multicellular electrophysiological interactions in intact biological tissue requires potent tools to measure and alter electrical behavior of interrogated cell populations in a controlled manner. In optogenetics, this can be implemented by cell-specific expression of fluorescent reporter proteins that are sensitive to changes in membrane voltage or cellular ion concentrations (pH, Ca^2+^), and by optogenetic actuators (mainly retinal-binding microbial opsins) that act as light-activated ion conductors in the targeted cells (Deisseroth et al., 2006; Miesenböck, 2009). Cation non-selective channelrhodopsins such as ChR2 mediate light-gated currents that depolarize excitable cells, including neurons and cardiomyocytes, beyond the threshold for action potential (AP) generation, thereby allowing optical pacing (Boyden et al., 2005; Bruegmann et al., 2010). Conversely, prokaryotic proton (e.g., ArchT), chloride (e.g., N_p_HR), and sodium (e.g., KR2*)* pumps can mediate outward photocurrents that drive membrane hyperpolarization (Han and Boyden, 2007; Zhang et al., 2007; Han et al., 2011; Inoue et al., 2013; Grimm et al., 2018). These light-driven ion pumps have been used to silence neuronal activity *in vitro* and *in vivo* (Zhang et al., 2007; Gradinaru et al., 2008; Arrenberg et al., 2009; Han et al., 2011; Kato et al., 2015; Grimm et al., 2018) and to slow down heart rate or to block conduction by selectively illuminating either pacemaker or atrioventricular canal tissue in the developing zebrafish heart (Arrenberg et al., 2010). A major limitation of hyperpolarizing ion pumps is their capacity to maximally transport one ion per absorbed photon, requiring high membrane expression levels of the pump in combination with high-intensity sustained illumination to effectively inhibit AP generation (Gradinaru et al., 2010; Zhang et al., 2011; Mattis et al., 2012). Moreover, by actively transporting ions against electrochemical gradients, prolonged pump activation alters cellular energy balance and trans-membrane ion distribution, with potentially unintended side-effects on other facets of cellular behavior (Raimondo et al., 2012; Alfonsa et al., 2015; Mahn et al., 2016).

The optogenetic toolbox has recently been extended by anion-selective channelrhodopsins (ACR). First variants (ChloC, iC1C2) were engineered by targeted mutagenesis of cation channelrhodopsins and showed a residual proton conductance (Berndt et al., 2014; Wietek et al., 2014), which was largely eliminated in subsequent optimized variants (iChloC, iC++) (Wiegert et al., 2015; Berndt et al., 2016). Engineered ACR have been complemented by naturally occurring ACR, first identified in the cryptophyte species *Guillardia theta* (GtACR1 and GtACR2) (Govorunova et al., 2015a), and more than 20 related proteins have been reported so far (Govorunova et al., 2015b, 2017; Wietek et al., 2016). Upon expression in animal cells, GtACR1 and GtACR2 support large Cl^−^-mediated currents with high operational light sensitivity (Govorunova et al., 2015a). GtACR1 and GtACR2 have been used in a number of studies to inhibit neuronal firing by “shunting” the membrane potential toward the reversal potential (*E*_*rev*_) for Cl^−^ (Malyshev et al., 2017; Mauss et al., 2017; Mohamed et al., 2017; Forli et al., 2018). First cardiac use of ACR was performed by Govorunova et al. comparing photocurrents of GtACR1 and the proton pump Arch3 in neonatal rat ventricular cardiomyocytes. GtACR1 required light intensities that were four orders of magnitude lower than those needed for Arch3 to activate photocurrents of comparable amplitudes. Using extracellular potential recordings, the authors observed that GtACR1-driven Cl^−^ currents reversibly inhibit spontaneous AP in cultured neonatal cardiomyocytes at light levels at which inhibition by Arch3 was inefficient (Govorunova et al., 2016).

In the current study, we characterize the electrophysiological and optical activation properties of GtACR1 in HEK 293T cells and primary cultured ventricular cardiomyocytes from rabbits. We assess the mechanism by which activation of GtACR1 may arrest cardiac AP and cellular contractions in rabbit isolated cardiomyocytes and in zebrafish hearts, both *ex* and *in vivo*. We show that GtACR1 enables large Cl^−^ currents that can be titrated by changing light intensity and/or light pulse duration. Using patch-clamp recordings, we observe depolarizing photocurrents at negative membrane potentials for all tested Cl^−^ concentrations. Using intracellular microelectrode recordings in zebrafish hearts, we confirm GtACR1-mediated depolarization of resting cells *in situ*. Thus, light-induced inhibition of cardiomyocyte excitability upon sustained GtACR1 activation is based on membrane depolarization. Short light pulses, in contrast, allow one to optically pace isolated cells and whole hearts, and to trigger cardiac contractions.

We conclude that ACR mediate depolarizing photocurrents in resting cardiomyocytes, which can be used for both activation and inhibition of cardiac activity. The observed inhibition is not based on reaching a physiological resting (re-/hyperpolarized) state. A suitable optogenetic tool to silence cardiomyocyte activation by clamping the membrane potential to diastolic resting potential levels by opening a K^+^ conductance has recently been reported (Bernal Sierra et al., 2018), but further effort will need to be applied to accelerating the system's on- and off-kinetics. A rapid K^+^-based silencing tool would be useful to temporarily block cardiac conduction in defined cell populations with the aim of investigating the basic mechanisms underlying atrial and ventricular arrhythmogenesis and cardioversion. In the future, this could facilitate the development of optimized strategies for arrhythmia termination, potentially by optogenetic approaches.

## Results

### Ion Selectivity of GtACR1

**Figure 1:**
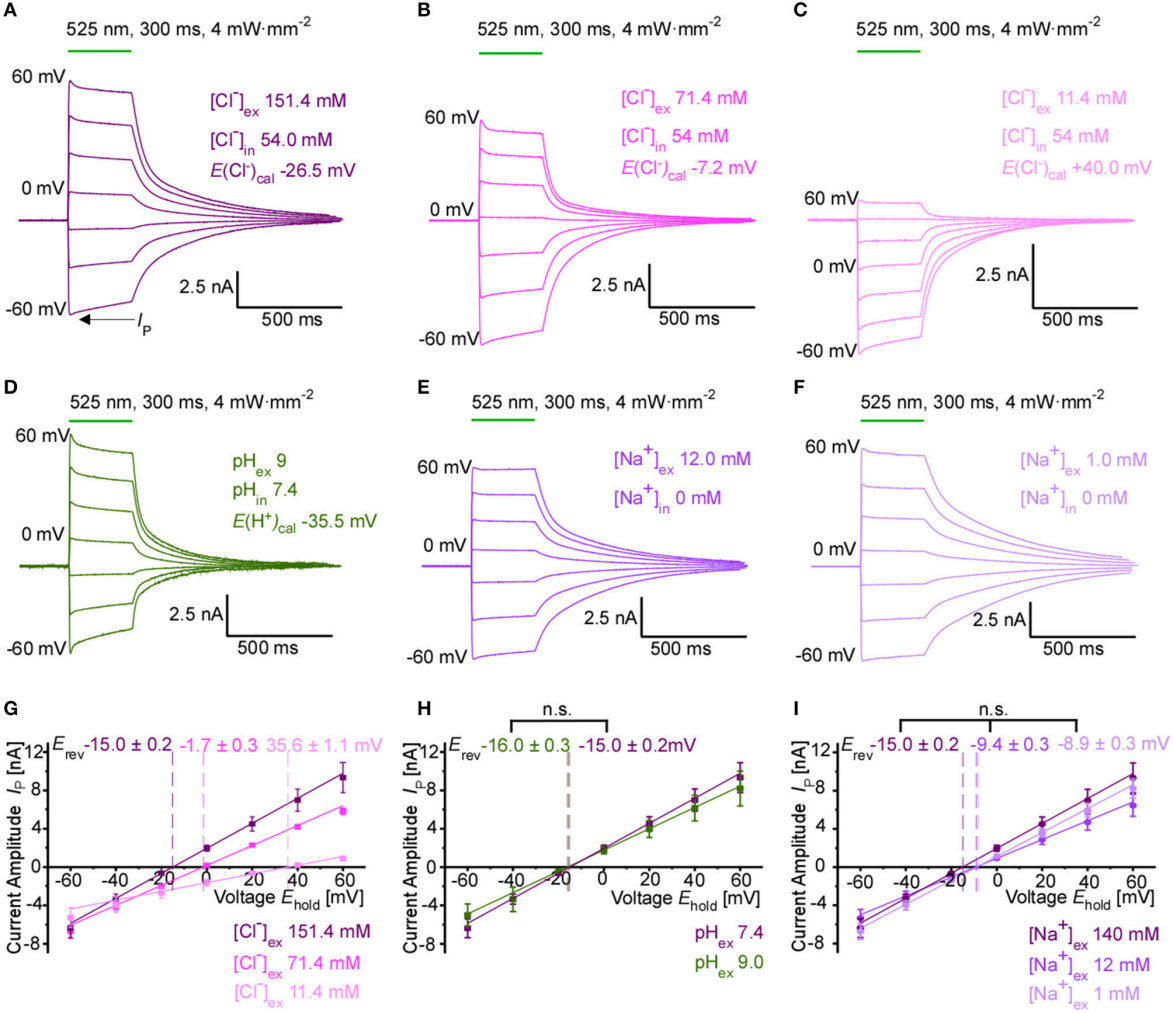
Ion selectivity of GtACR1-driven photocurrents in HEK 293T cells. **(A–F)** Representative whole-cell currents at holding membrane potentials from −60 mV to +60 mV (step size 20 mV). Peak current *I*_P_ is indicated. The calculated reversal potential *E*_rev, cal_ is stated at 25°C. Standard pipette solution contained 54 mM Cl^−^ and 0 mM Na^+^. External bath solution contained **(A)** standard high, **(B)** moderately reduced and **(C)** strongly reduced Cl^−^ concentration. (**D**) Photocurrent using an alkaline external solution (pH 9.0), Cl^−^ concentrations as in **(A)**. **(E,F)** Photocurrent at moderately reduced **(E)** and strongly reduced Na^+^ concentration **(F)**, Cl^−^ concentrations as in **(A)**. **(G)** *IE* relationship recorded at different external Cl^−^ concentrations (*n* = 8–12). Mean reversal potentials (*E*_rev_) ± SE are marked in the respective color. **(H)** *IE* relationship recorded at pH 7.4 and pH 9.0 (*n* = 8–12, Mann-Whitney *p* > 0.05). **(I)** *IE* relationship recorded at various Na^+^ concentrations (*n* = 10–12, Mann-Whitney *p* > 0.05).

We performed whole-cell patch-clamp recordings in transiently transfected HEK 293T cells to characterize in detail the GtACR1-mediated photocurrents and their ion dependency. Upon green-light application, GtACR1 activation results in large photocurrents whose direction and amplitude depends on the Cl^−^ concentration gradient (Figures 1A–C). *E*_*rev*_ for GtACR1-mediated currents are −15.0 ± 0.3, −1.7 ± 0.2, and +35.6 ± 1.1 mV for extracellular Cl^−^ concentrations ([Cl^−^]_ex_) of 151.4, 71.4, and 11.4 mM, respectively, (with an intracellular Cl^−^ concentration [Cl^−^]_in_ of 54.0 mM) (Figure 1G). The experimentally observed reversal potentials deviate from calculated reversal potentials for a pure chloride channel at 25 °C in a non-linear manner (theoretical reversal potentials according to the Nernst equation are −26.5, −7.2, and +40.0 mV, respectively). Decreasing Cl^−^ levels of the internal (pipette) solution shifts reversal potentials to more negative values (−32.1 ± 1.0 and −45.8 ± 2.8 mV at 15 and 4 mM [Cl^−^]_in_, respectively; Supplementary Figure 1A), again with major deviations from theoretically predicted potentials for a pure Cl^−^ conductance (−59.4 and −93.4 mV, respectively). For all Cl^−^ concentration gradients tested, we observe inward currents (in line with a net outward movement of anions) at negative membrane potentials. To test for a potential additional cation conductance that would explain differences between theoretical and experimentally observed reversal potentials, we also varied external pH, K^+^ and Na^+^ concentrations. Photocurrent amplitudes and reversal potentials are not altered when reducing external proton concentration to pH 9.0 (Figures 1D,H) or varying external K^+^ concentration (Supplementary Figure 1B). Decreasing the extracellular Na^+^ concentration shifts the observed whole-cell current reversal potential to slightly more positive values (−9.4 ± 0.3 and −8.9 ± 0.3 mV at 12 and 1 mM [Na^+^]_ex_, respectively; [Cl^−^]_in_ of 54.0 mM), while average current amplitudes at negative membrane potentials appear unaffected (Figures 1E,F,I). Similar changes in reversal potential by variation of the internal Cl^−^ concentration, and the absence of a sensitivity to pH were confirmed in cardiomyocytes expressing GtACR1 (Supplementary Figures 1C,D).

### Photoactivation of GtACR1 in Cardiomyocytes

**Figure 2:**
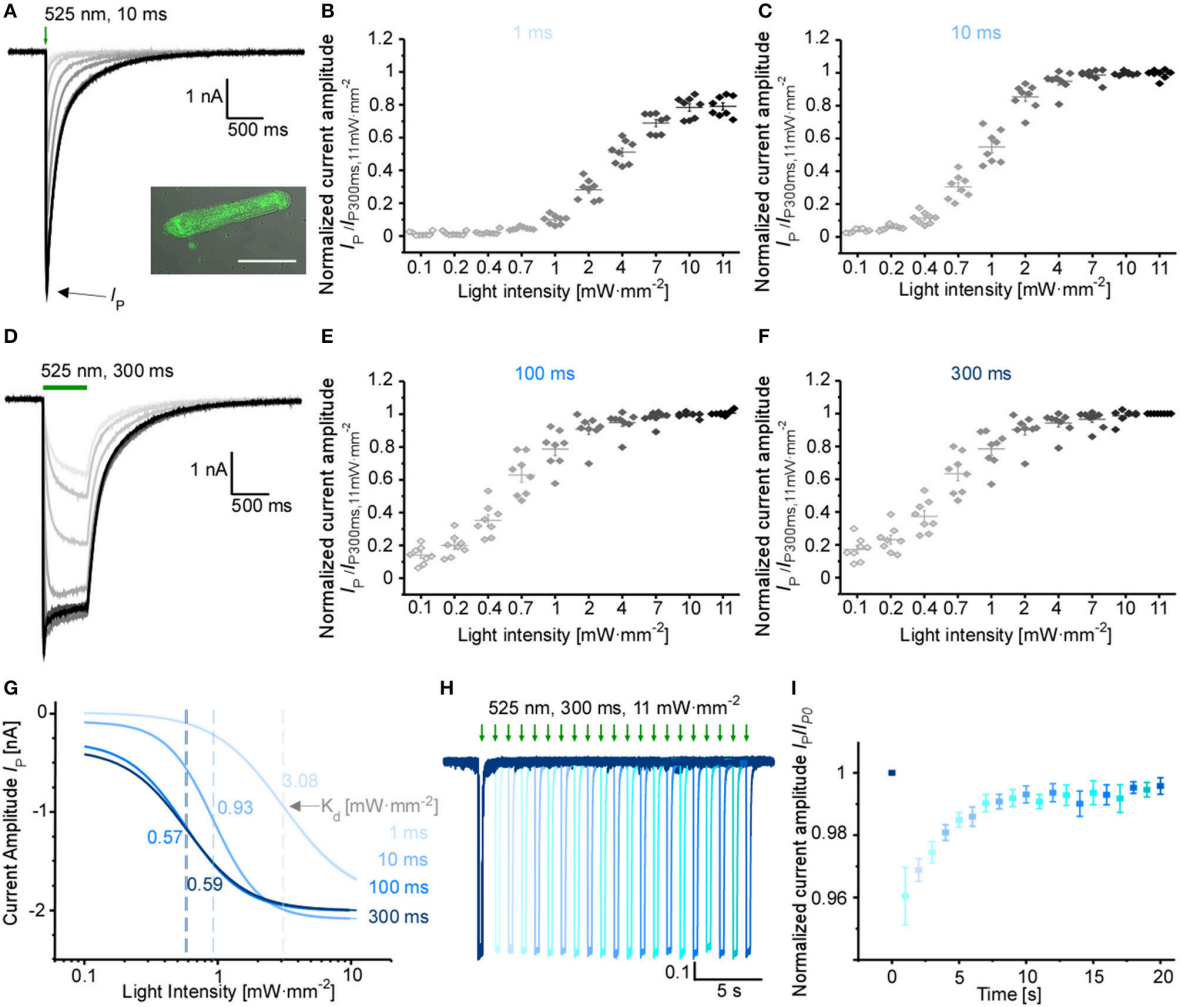
Characterization of GtACR1 currents in rabbit isolated cardiomyocytes. **(A)** Representative voltage-clamp recordings at −60 mV using light pulses of 10 ms. The insert shows a fluorescence image of GtACR1-eGFP expressed in cultured cardiomyocytes. Scale bar 50 μm. **(B,C,E,F)** Light titration at various light intensities using light pulses of **(B)** 1 ms, **(C)** 10 ms, **(E)** 100 ms, **(F)** 300 ms (*n* = 8, *N* = 2). Please note non-linear scaling of x-axis. **(D)** Representative voltage-clamp-recordings at −60 mV using light pulses of 300 ms. **(G)** Mean peak current *I*_P_ was fitted by the Hill equation. Dissociation constant K_d_ [mW·mm^−2^] was calculated for each light duration (K_d, 1ms_ = 3.08 ± 0.17, K_d, 10ms_ = 0.93 ± 0.03, K_d_, _100ms_ = 0.57 ± 0.04, K_d, 300ms_ = 0.59 ± 0.04). **(H)** Representative voltage-clamp recording at −60 mV. Each trace was normalized to the first peak. Dark interval of Δ1 s between light pulses in each dual-stimulation experiment. **(I)** Recovery of normalized peak current (*n* = 7, *N* = 2) during repeat optical stimulation, measured at maximum light intensity of 11 mW·mm^−2^ and light pulse duration of 300 ms.

Using adenoviral transduction, GtACR1-eGFP was expressed in cultured primary ventricular cardiomyocytes from rabbit hearts (Figure 2A insert). We performed whole-cell patch clamp recordings to test the functionality of the Cl^−^ channel in cardiomyocytes. At a holding potential of −60 mV, green light pulses elicit large inward currents that can be graded by applied light intensity and/or pulse duration (Figures 2A–G). Half-maximal peak amplitudes are reached at 3.08 and 0.93 mW·mm^−2^ for short light pulses of 1 and 10 ms, respectively. Using longer light pulses (≥100 ms) half-maximal amplitudes are observed at approximately 0.58 mW·mm^−2^. Channel closure follows a bi-exponential decline after illumination terminates (see Table 1 for τ values for selected exposure times and light intensities). When activated by longer light exposure, a proportion of channels inactivates (or transitions to an open state of lower conductance) resulting in stationary currents of reduced amplitude (Figure 2D). Repetitive channel activation results in transient channel inactivation reflected by reduction of the peak current (*I*_p_) by 4%, which recovers to initial values with τ_recovery_ = (2.2 ± 0.2) s (Figures 2H,I). Our data provides evidence that GtACR1 is functional in cardiomyocytes without addition of external retinal and that current characteristics can be titrated using light pulses of different intensity and duration.

**Table 1:** Bi-exponential current decay after light activation of GtACR1 in ventricular cardiomyocytes.

**Time constants at −60 mV**	**Low light intensity (0.1 mW·mm**^**−2**^)		**High intensity (4 mW·mm**^**−2**^)	
	**τ_off1_ (ms)**	**τ_off2_ (ms)**	**τ_off1_ (ms)**	**τ_off2_ (ms)**
Short light pulses (10 ms)	32 ± 2	125 ± 19	46 ± 3	224 ± 18
Prolonged light pulses (300 ms)	36 ± 1	172 ± 14	48 ± 4 23 ± 3	271 ± 23 246 ± 15

*Black values were obtained at −60 mV (n = 8); gray values reflect current decline at +20 mV (n = 20)*.

### AP Inhibition Using Prolonged GtACR1 Activation *in vitro*

**Figure 3:**
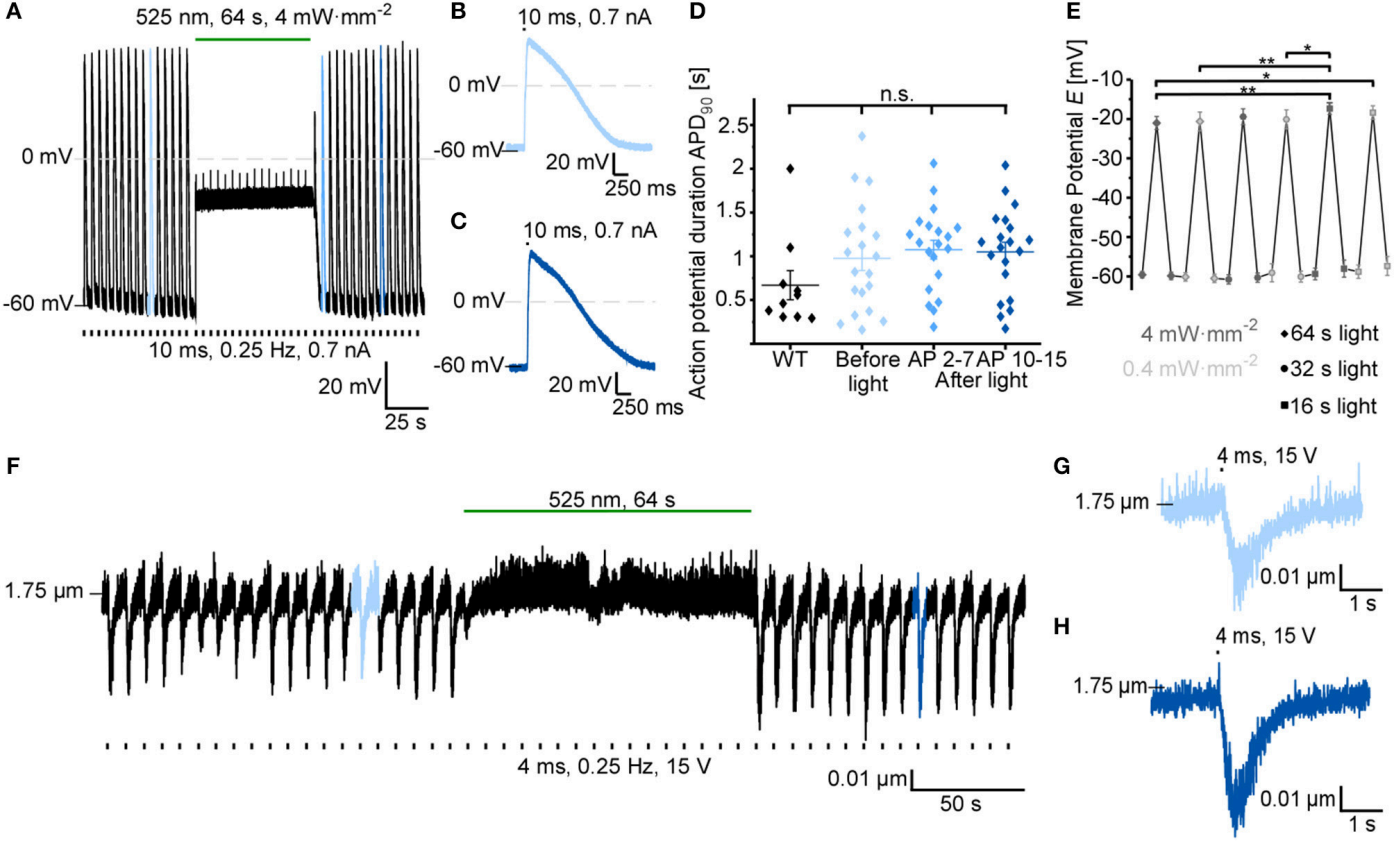
GtACR1 inhibits electrically paced cardiomyocytes by depolarization. **(A)** Representative trace of cardiomyocyte membrane potential during electrical pacing (EP) at 0.25 Hz (current injections of 0.7 nA for 10 ms). A sustained 64 s light pulse inhibits AP generation by depolarizing the cell to about −20 mV. **(B)** 10^th^ AP before light application. **(C)** 10^th^ AP after end of light exposure. **(D)** Comparison of APD_90_ before light (AP 2-7) and at two different time points after light exposure (average APD_90_ of AP 2-7 and AP 10-15) to APD_90_ in non-transduced WT cells (WT *n* = 10, *N* = 1; GtACR1 transduced cells *n* = 23, *N* = 2). Wilcoxon test (WT vs. before light): *p* > 0.05. ANOVA one-way repeated Dunnett test (before vs. after light): *p* > 0.05. **(E)** Membrane potential *E* was recorded before, during and after light pulses of various durations (64 s, 32 s, 16 s) and light intensities (4 mW·mm^−2^, 0.4 mW·mm^−2^; *n* = 11, *N* = 2). Results of ANOVA one-way repeated Tukey are indicated in graph (*p ≤ 0.05, **p ≤ 0.01). **(F)** Representative sarcomere length recording. The cell was electrically paced at 0.25 Hz by field-stimulation (15 V) and sarcomere length shortening was inhibited by light for 64 s. **(G)** 6^th^ contraction before light application. **(H)** 10^th^ contraction after light application.

Based on previous reports using GtACR1 for neuronal and cardiac silencing, we assessed the utility of using GtACR1 to inhibit electrically evoked AP in ventricular cardiomyocytes. In current-clamp recordings, AP are triggered by 10-ms current ramps at 0.25 Hz. Prolonged green light application (64 s at 4 mW·mm^−2^) results in membrane depolarization (to −21.0 ± 1.7 mV), thereby preventing further AP generation (Figure 3A). Light-induced inhibition is reversible, and AP before and after optical silencing show comparable durations at 90% repolarization (APD_90_; Figures 3B–D). Using an illumination protocol with varying pulse duration and light intensity for repeated depolarization of cardiomyocytes, we found that the average membrane potential during repeated light exposure increases to more positive values over time (starting membrane potential: −59.6 mV, end membrane potential: −57.4 mV). This may be due to changes in intracellular ion concentrations (Figure 3E, for exemplary current traces see Supplementary Figure 2). In contrast, green light application does not affect resting membrane potential and APD_90_ in non-transduced control cells (Supplementary Figures 1G,H). Light-induced silencing of cardiomyocytes was also assessed in intact cells by measuring sarcomere length changes during electrical field stimulation. Similar to electrophysiological recordings, sustained light inhibits cardiomyocyte activity (Figure 3F). Upon onset of sustained illumination, we observed a short and low-amplitude contraction. While resting sarcomere length is slightly reduced in GtACR1-expressing cells compared to non-transduced control cells, no differences were observed in fractional sarcomere shortening or contraction kinetics before and after light-induced silencing compared to control cells (Figures 3G,H, Supplementary Figures 1I–N).

### GtACR1-Mediated Optical Pacing *in vitro*

**Figure 4:**
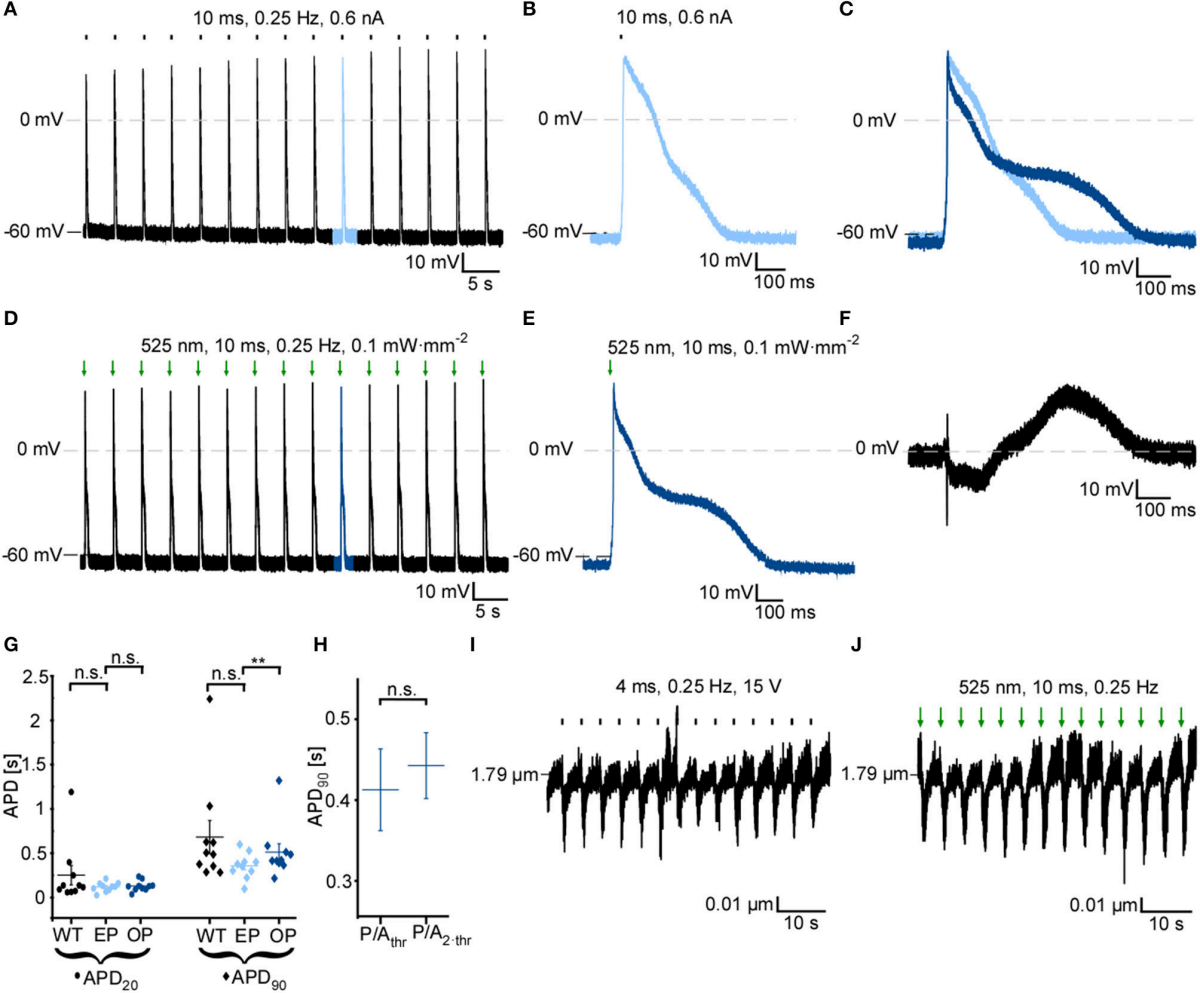
GtACR1 activation by short light pulses enables optical pacing of cardiomyocytes. **(A)** Representative current-clamp recording at 0 pA during electrical pacing (EP) at 0.25 Hz using a current ramp of 0.6 nA, 10 ms. **(B)** 10^th^ AP of electrically paced cardiomyocyte. **(C)** Overlay of the 10^th^ AP of electrically (light blue) and optically (dark blue) paced cardiomyocyte. AP were aligned by d*E*/d*t*_max_. **(D)** Representative current-clamp recording at 0 pA during optical pacing (OP) at 0.25 Hz by light pulses of 525 nm, 10 ms, 0.1 mW·mm^−2^. **(E)** 10^th^ AP of optically paced cardiomyocyte. **(F)** Difference of membrane potential during AP [shown in **(C)**] between OP and EP (*E*_OP_-*E*_EP_). **(G)** Comparison of AP duration of GtACR1-expressing cardiomyocytes during OP and EP to electrical stimulation of non-transduced WT cells (*n* = 10, *N* = 2). Mean APD_20_ and APD_90_ of the 10^th^ to 15^th^ AP are compared. Wilcoxon test (EP vs. OP): APD_20_ *p* > 0.05, APD_90_ *p* < 0.01, Mann-Whitney test (WT vs. EP): APD_20_ *p* > 0.05, APD_90_ *p* > 0.05. **(H)** APD_90_ of optically paced cardiomyocytes at threshold illumination (light power per area; *P*/*A*_thr_) and two-times threshold light intensity (*P*/*A*_2·*thr*_). Wilcoxon test: *p* > 0.05 (*n* = 6, *N* = 1). **(I)** Sarcomere length recording of representative cell. Cardiomyocyte was electrically paced by field stimulation at 15 V, 0.25 Hz. **(J)** Sarcomere length recording of representative cell. OP by light pulses of 10 ms, 0.25 Hz. Statistical tests: n.s. *p* > 0.5, **p ≤ 0.01.

Since GtACR1 activation leads to cardiomyocyte membrane depolarization at negative membrane potentials, we tested whether GtACR1-expressing cardiomyocytes can be optically paced. Short low-intensity light pulses (10 ms, 0.1 mW·mm^−2^) are sufficient to reliably trigger AP in cultured GtACR1-positive cells (Figures 4D,E), but not in wild-type controls. When compared to electrically induced AP (Figures 4A–G), optically stimulated AP show prolonged late repolarization, reflected by a significantly longer APD_90_, while APD at 20% repolarization (APD_20_) is not different between optical (OP) and electrical (EP) pacing. Also, optically triggered AP show a slower onset and a late depolarized phase, as seen in the voltage difference plot between optically and electrically stimulated AP shown in Figure 4F. Diastolic membrane potential was slightly increased (more positive) with EP compared to OP (Supplementary Figure 1E). Resting membrane potential and APD_90_ with OP are not altered when light intensity is increased from threshold to 2 × threshold (Figure 4H, Supplementary Figure 1F). Finally, in experiments tracking sarcomere length changes in intact cells, we observe successful OP, leading to cardiomyocyte contractions similar to electrically triggered beats (Figures 4I,J).

### Effects of Prolonged GtACR1 Activation in Intact Zebrafish Hearts

Zebrafish served as a model system to study effects of GtACR1 activation in native myocardium (Rafferty and Quinn, 2018; Ravens, 2018). GtACR1-eGFP was expressed specifically in cardiac myocytes in the hearts of zebrafish using Tol2 transposon transgenesis by microinjection of a cmlc2:GtACR1-eGFP plasmid at the one-cell stage. Fish with successful gene insertion, indicated by cardiac eGFP expression, were raised to 3 months. Hearts were isolated and intracellular microelectrode recordings of ventricular myocyte membrane potential were performed.

**Figure 5:**
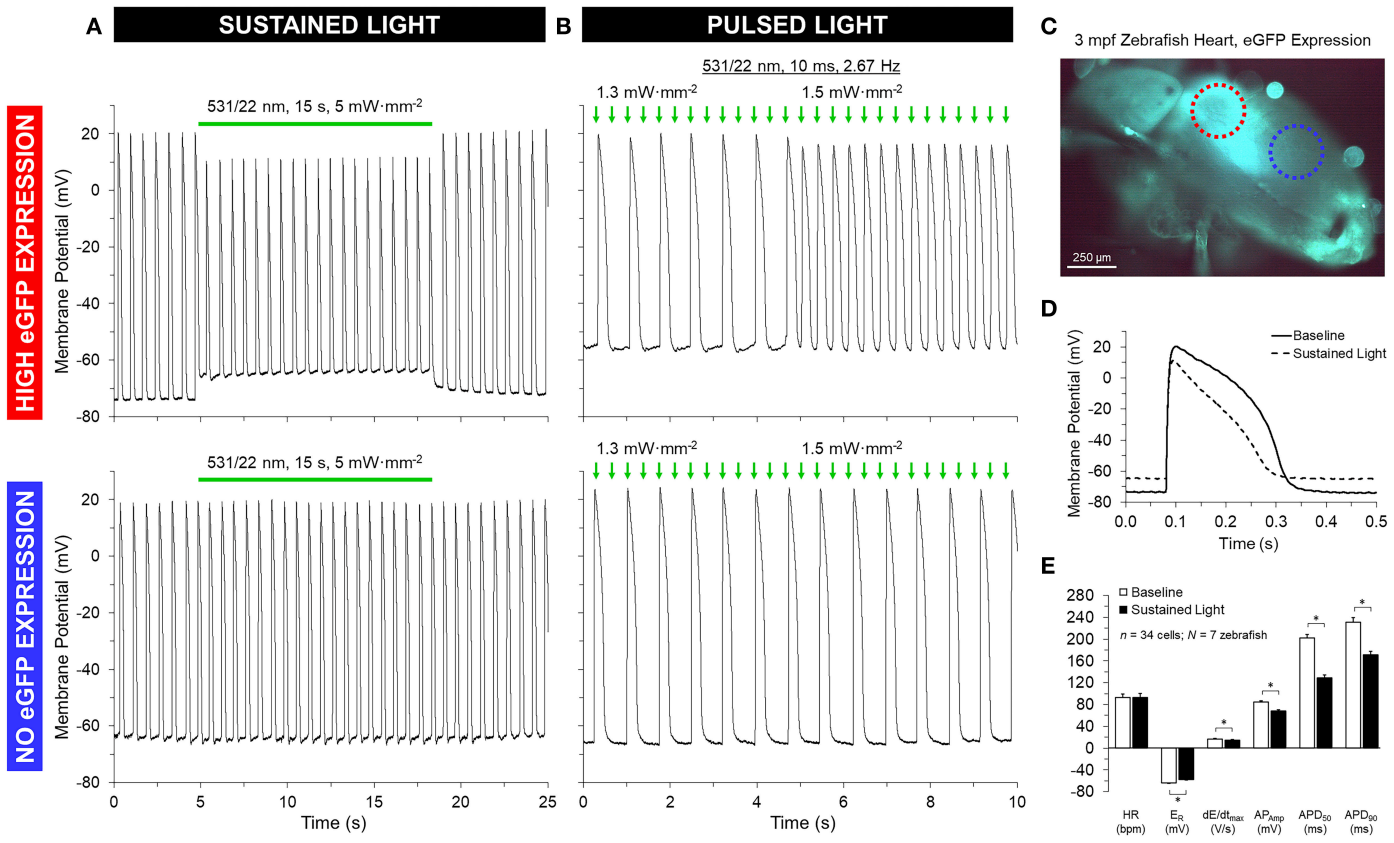
GtACR1 activation depolarizes ventricular myocyte membrane potential in isolated zebrafish hearts. Regions of high or no eGFP expression on the ventricle of 3-months post fertilization (mpf) zebrafish isolated hearts were illuminated by a 0.16 mm^2^ spot of 531/22 nm light sustained for 15 s or pulsed for 10 ms at a rate 2–3 × sinus heart rate. **(A,D–E)** In the case of sustained light, in regions displaying eGFP expression (red circle in **C**) there was an immediate increase in resting membrane potential (*E*_R_) and a decrease in the maximum rate of membrane depolarization (d*E*/d*t*_max_), AP amplitude (AP_Amp_), and APD at 50% and 90% repolarization (APD_50_ and APD_90_). **(B)** In the case of pulsed light, the heart could be stimulated when light intensity was increased to supra-threshold values. **(A,B)** In both cases, there was no effect seen in regions displaying no eGFP expression (blue circle in **C**). *indicates *p* < 0.0001 by two-tailed paired Student's *t*-test.

**Figure 6:**
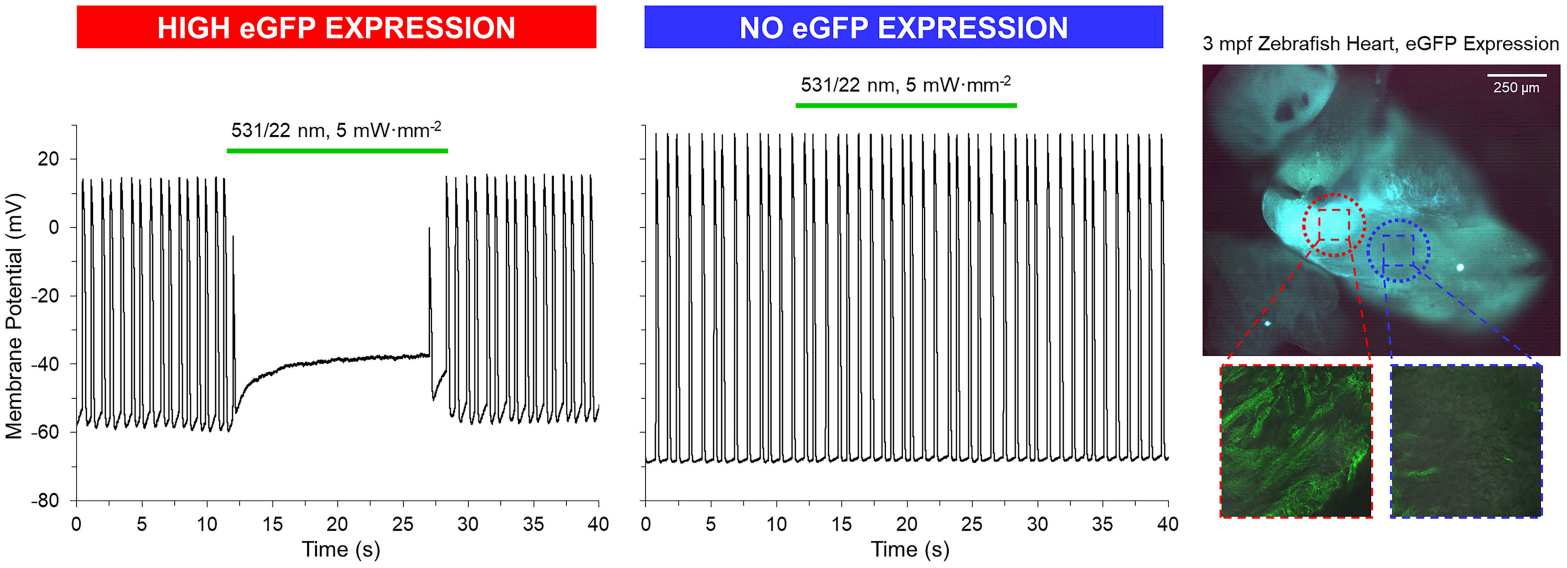
GtACR1 activation can inhibit ventricular myocyte action potentials in isolated zebrafish hearts. In a subset of 3-months post fertilization (mpf) isolated zebrafish hearts (*n* = 3/7), a 0.16 mm^2^ spot of 531/22 nm light applied to some eGFP expressing regions locally inhibited AP by depolarization of membrane potential, while there was no effect in regions with no eGFP expression (confirmed by live confocal microscopy).

GtACR1-eGFP expression in the ventricle was largely epicardial and heterogeneous at the cellular level (Supplementary Figure 3), with regions of high eGFP expression next to eGFP-free areas at the whole-heart level (Figures 5, 6). Light for activation of GtACR1 was focused on regions of high or no eGFP expression (spot size of 0.16 mm^2^). When sustained light (5 mW·mm^−2^) is applied to regions of high eGFP expression, there is an immediate depolarization of resting membrane potential (*E*_R_) and a decrease in the maximum rate of membrane potential change (d*E*/d*t*_max_) during the AP upstroke, as well as in AP amplitude (AP_Amp_), APD_50_ and APD_90_ (Figure 5, *n* = 34 cells, *N* = 7 zebrafish). Additionally, in regions of high eGFP-expression in some hearts (*N* = 3/7), sustained light locally inhibits AP generation by depolarization of membrane potential (Figure 6). Ventricular contractions could also be inhibited *in vivo* by exposing the entire ventricle (spot size of 0.05 mm^2^) of intact eGFP-positive zebrafish larvae (21 days post fertilization, *N* = 3) to sustained light (1.7 mW·mm^−2^; Supplementary Movie 1).

### Optical Pacing of Zebrafish Hearts

When pulsed light (10 ms, 2 × sinus heart rate) of supra-threshold intensity (1.2 ± 0.2 mW·mm^−2^; determined in each heart by increasing light intensity until AP stimulation occurred) is applied to regions of high eGFP expression, AP are elicited, resulting in OP of the heart (Figure 5). Comparison of AP, stimulated by OP and EP at the same rate, shows no difference in AP characteristics (Supplementary Figure 4). As with sustained light, pulsed light also had an effect *in vivo* in intact eGFP-positive zebrafish larvae, eliciting ventricular contractions resulting from OP. In contrast to GtACR1-expressing regions, neither sustained nor pulsed light application to eGFP-negative myocardial tissue in the same animals, or to ventricular myocardium from wild-type zebrafish (*N* = 3), induces any changes in membrane potential.

## Discussion

Tools to modulate cardiac electrophysiology in a spatio-temporally defined, cell-specific manner hold a key to improving our understanding of electrophysiological signaling in health and disease. ChR2 can be used for optical pacing, and for inhibition of AP generation—albeit by non-physiological sustained depolarization of the resting membrane potential. Prolonged membrane depolarization will lead to ion concentration imbalances, which could be particularly problematic in diseased tissue (Schneider-Warme and Ravens, 2018). ACR have been used to inhibit AP generation in neuronal cell populations and neonatal rat ventricular cardiomyocytes (Govorunova et al., 2016; Malyshev et al., 2017; Mauss et al., 2017; Mohamed et al., 2017; Forli et al., 2018). Here, we investigate their use in rabbit cardiomyocytes, and in intact zebrafish hearts.

We show that green-light activation of GtACR1 triggers depolarizing photocurrents in resting cardiomyocytes. GtACR1 currents are based on Cl^−^ conductance, with only minor current contributions by cations, such as Na^+^, K^+^, or H^+^. Accordingly, cardiomyocyte inhibition by sustained illumination is based on polarizing cells toward the reversal potential for Cl^−^. In line with an estimated reversal potential between −40 and −33 mV in cardiac myocytes (Clemo et al., 1999; Baumgarten et al., 2005), GtACR1-mediated myocyte inhibition relies on membrane depolarization, thus preventing myocyte repolarization. While keeping cardiomyocytes at a depolarized potential can block re-excitation and conduction, sustained depolarization will result in Ca^2+^ and Na^+^ overload *via* activation of additional ion fluxes, e.g., through L- and T-type Ca^2+^ or background Na^+^ channels. This would be detrimental in cases where cells are already overloaded with Ca^2+^ or Na^+^, e.g., when using GtACR1 for inhibition of cardiomyocyte activity to terminate arrhythmias in pathologically remodeled myocardium (note: thus far, all experiments studying depolarization-based optogenetic inhibition have used “healthy” cardiomyocytes as a model system).

In contrast to cardiomyocytes, the reversal potential for Cl^−^ is closer to the resting membrane potential in the somatodendritic compartment of neurons, in which ACR have a hyper- or re-polarizing effect. Thus, ACR activation mimics neuronal inhibition by postsynaptic GABA_A_ receptors (Wiegert et al., 2017). It would be wrong, though, to assume that ACR necessarily hyperpolarize all resting cells—as shown here. There are neuronal compartments with elevated intracellular chloride concentrations, including presynaptic terminals and axons, where ACR activation can lead to depolarizing photocurrents. In fact, several studies have reported ACR-triggered presynaptic vesicle release and antidromic spikes (Mahn et al., 2016; Malyshev et al., 2017). To overcome these excitatory effects, soma-targeted ACR have been constructed by addition of the soma-targeting motifs of the K^+^ channel K_v_2.1 and telencephalin (Mahn et al., 2018; Messier et al., 2018). While optical inhibition with soma-targeted ACR can be effective in neurons, ACR-based cardiomyocyte inhibition is based on non-physiological sustained depolarization, as shown here both for isolated cells and myocytes within intact myocardium. A light-gated K^+^ conductance could serve as a potent alternative for optical inhibition of excitatory cells (Bernal Sierra et al., 2018), as the resting potential of excitable cells is close to the K^+^ reversal potential, thereby limiting secondary ion fluxes.

Based on depolarizing photocurrents, GtACR1 enables OP of cardiomyocytes *in vitro* and in zebrafish ventricles both *ex* and *in vivo*. In isolated myocytes, optically stimulated AP show delayed late repolarization, compared to electrically triggered AP. This can be explained by (i) the slow component of GtACR1 closing kinetics, resulting in residual photocurrents even after 200 ms (Table 1) and (ii) secondary activation of intrinsic Ca^2+^ channels (e.g., L-type Ca^2+^ channel) and/or the Na^+^/Ca^2+^ exchanger in forward mode. Regarding photocurrent decline after illumination, one should take into account that channel closure is usually decelerated at more depolarized membrane voltages in channelrhodopsins, and only few variants (ChR2 T159C E123T; Chronos) display more or less voltage-insensitive closing kinetics (Schneider et al., 2015). In line with our data (Table 1), GtACR1 has been reported to show biphasic channel closure, with the fast component accelerating at depolarized potentials, whereas the slow component is similar at both positive and negative membrane voltages (Sineshchekov et al., 2015). To prevent prolongation of optical triggered AP, one could use an ACR with faster closing kinetics, such as the GtACR1-C237A mutant (Kim et al., 2018), PsACR1 (Govorunova et al., 2015b; Wietek et al., 2016) or ZipACR (Govorunova et al., 2017). In ventricular cells of intact zebrafish hearts, optically evoked AP display comparable amplitude and kinetics to same-frequency electrically stimulated AP. This difference between isolated and *in situ* myocytes may reflect effects of electrotonic coupling between GtACR1-expressing and non-expressing cells in the zebrafish heart. Indeed, confocal microscopy revealed heterogeneous GtACR1 expression at the cellular level, even in high eGFP-expression regions of zebrafish hearts, with direct proximity of expressing and non-expressing cells. Electrotonic effects may also explain the less pronounced GtACR1-mediated membrane depolarization observed in isolated zebrafish hearts, compared to rabbit isolated myocytes. Future studies with an established transgenic zebrafish line in which GtACR1-eGFP is expressed in myocytes homogeneously across the heart (generated by in-crossing the micro-injected fish used for testing in this study) will allow this hypothesis to be tested.

While ACR do not allow hyperpolarization of cardiomyocytes, they may be used to mimic Cl^−^ currents in cardiac cells. Both inward and outward chloride currents are present during the cardiac AP cycle and at least six families of Cl^−^ channels are functionally expressed in cardiomyocytes. Cl^−^ channels have been found to play a role in cardiac arrhythmogenesis, myocardial hypertrophy and heart failure, as well as in cardioprotection against ischemia reperfusion (Duan, 2013). ACR may be used to investigate the underlying roles of Cl^−^ channels in these disease settings, with light not only allowing for precise spatiotemporal control of ACR activity, but also enabling titration of current amplitudes by variation of light intensity and/or duration. Cell-type specific gene targeting may further enable investigating effects of Cl^−^ conductance on cells other than cardiomyocytes, and might be useful for studying heterocellular interactions between cardiomyocytes and non-myocytes (Johnston et al., 2017). Recent studies expressing optogenetic probes specifically in either cardiac macrophages of healthy mouse hearts, or in non-myocytes of ventricular scar-border zone tissue, showed functional electronic coupling of the targeted cell types to adjacent cardiomyocytes (Quinn et al., 2016; Hulsmans et al., 2017). The relevance of such coupling, however, both in normal homeostasis and in the context of cardiac tissue remodeling, remains to be determined. Optogenetic perturbation of electronically coupled non-myocytes might be a useful approach here, for example to modulate cardiac electrophysiological properties such as conduction velocity without interfering with cardiomyocyte function (as pioneered by Hulsmans et al., 2017).

Using GtACR1 to study cardiac electrophysiology in human tissue or hearts from larger mammals is limited by the restricted tissue penetration of green light needed for GtACR1 activation (maximal activation at ~515 nm). This could be overcome by using flexible micro-LED systems for local light delivery (Ayub et al., 2017), or by replacing GtACR1 with a red-shifted ACR. Recent studies have identified ACR with action spectra shifted to longer wavelengths, both by protein engineering (Wietek et al., 2017) and genomic screening (Govorunova et al., 2017). The recently reported crystal structure of GtACR1 will facilitate further spectral tuning, e.g., by transferring mutations identified in the most red-shifted channelrhodopsin variant Chrimson S169A to GtACR1 and related ACR (Kim et al., 2018; Oda et al., 2018). Computational modeling has predicted that epicardial (and even pericardial) illumination will be sufficient for transmural activation of red-light gated channelrhodopsins in human hearts. This model considered the less light-sensitive ChR2, under the assumption of activation at 669 nm at an intensity of 10 mW·mm^−2^ (4.7-fold higher for pericardial light delivery) (Bruegmann et al., 2016). Thus, on the premise of further optimized means of light delivery and tuned rhodopsin variants, it should become feasible to use ACR for triggering transmural Cl^−^ currents in hearts from larger animals. Application to humans would require addressing a host of other issues, such as targeting specificity and avoidance of immunogenic responses. These aspects are the subject of ongoing studies in the field.

In summary, GtACR1 represents a potent tool for light-induced depolarization of defined cardiac cell populations. As is the case for ChR2, this can be used both to activate and to silence AP generation in cardiomyocytes. Compared to ChR2, ACR shows only weak intrinsic cation conductance, rendering ACR a superior tool for some applications, such as OP of myocardium. One has to keep in mind, however, that sustained membrane depolarization for cardiac silencing will give rise to secondary ion fluxes that may affect transmembrane ion distribution. This could increase arrhythmogenicity in myocardium, and be particularly counter-productive when using optogenetic approaches in ischemic myocardium where cardiomyocytes are already overloaded with Na^+^ and/or Ca^2+^.

## Materials and Methods

If not stated otherwise chemicals were purchased from Sigma-Aldrich (St. Louis, MO, United States).

### Molecular Biology

pUC57-GtACR1 was a kind gift from Dr. Jonas Wietek, Humboldt-Universität zu Berlin. The codon-optimized (mouse, human) sequence encoding for GtACR1 (*GenBank* accession AKN63094.1) was subcloned into the peGFP-N1 vector (Clontech, Mountain View, CA, United States) using FastDigest NheI and AgeI (Thermo Fisher Scientific, Waltham, MA, USA).

For expression in cardiomyocytes, GtACR1-eGFP was cloned into pAdeno-CMV following the manufacturer's instructions (Adeno-X Adenoviral System, Clontech). Adenovirus was produced by the Viral Core Facility of the Charité—Universitätsmedizin Berlin, Germany.

### HEK 293T Cell Culture

HEK 293T cells were seeded onto coverslips at a final density of 25,000 cells/mL. After 24h, jetPEI Transfection Reagent (Polyplus transfection, Illkirch, France) was used for transfecting GtACR1-eGFP into HEK 293T cells. HEK cells were measured 24–48 h post-transfection.

### Cell Isolation and Culturing of Primary Cardiomyocytes

All rabbit experiments were carried out according to the guidelines stated in Directive 2010/63/EU of the European Parliament on the protection of animals used for scientific purposes and approved by the local authorities in Baden-Württemberg (X-16/10R). Culturing of isolated ventricular cells from rabbit hearts was performed as previously described (Bernal Sierra et al., 2018). Rabbits (9-10 weeks old of both sex [3 male, 4 female], total *N* = 7) were anesthetized via intramuscular injection of esketamine hydrochloride (Ketanest®S 25 mg/mL, Pfizer Pharma PFE GmbH, Berlin, Germany; 12.5 mg/kg body weight) and xylazine hydrochloride (Rompun® 2%, Bayer Vital GmbH, Leverkusen, Germany; 0.2 mL/kg body weight). During anesthesia 1,000 units heparin (Heparin-Sodium 5,000 I.E./mL, B. Braun Melsungen AG, Melsungen, Germany) and 5 mg esketamine hydrochloride were given intravenously. Thiopental (Thiopental Inresa 0.5 g, Inresa Arzneimittel GmbH, Freiburg, Germany; 12.5 mg/mL) was injected intravenously until apnea. The heart was excised and rinsed in normal Tyrode solution (Table 2). The aorta was cannulated, the heart was transferred to a Langendorff perfusion setup and perfused with normal Tyrode solution to wash out the blood. The heart was then perfused with Ca^2+^-free cardioplegic solution (Table 2) for 3 more minutes after the heart had stopped beating, and it was then digested with enzyme solution (Table 2) until the tissue appeared soft (45–60 min). Left ventricle and septum were separated and cells were released by mechanical dissociation in blocking solution (Table 2).

**Table 2:** Composition of solutions used for cell isolation.

	**Normal Tyrode solution**	**Ca^**2+**^- free cardioplegic solution**	**Enzyme solution**	**Blocking solution**
NaCl [mM]	137	137	137	137
KCl (VWR International bvba, Leuven, Belgium) [mM]	4	14	14	14
HEPES [mM]	10	10	10	10
Creatine [mM]	10	10	10	10
Taurine [mM]	20	20	20	20
Glucose [mM]	10	10	10	10
MgCl_2_ [mM]	1	1	1	1
Adenosine [mM]	5	5	5	5
L-carnitine [mM]	2	2	2	2
CaCl_2_ (Honeywell Fluka^TM^, Seelze, Germany) [mM]	1	-	0.1	0.1
Heparin-sodium [units/L]	5,000	-	
EGTA (Carl Roth GmbH, Karlsruhe, Germany) [mM]		0.096	0.096	0.096
Collagenase type 2, 315 U/mg (Worthington, Lakewood, NJ, USA) [g/L]			0.6
Protease XIV [g/L]			0.03
Bovine serum albumin [%]				0.5

*All solutions were adjusted to pH 7.4 at 37°C*.

After digestion cell suspensions were filtrated through a mesh (pore size [1 mm^2^]) and centrifuged for 2 min at 22 × *g* (gravitational acceleration). Fibroblasts remaining in the supernatant were removed and cardiomyocytes were resuspended in blocking solution. After cardiomyocytes settled, the supernatant was removed and cells were resuspended in plating medium (in mM: 5 creatine, 2 L-carnitine hydrochloride, 5 taurine, 1 sodium pyruvate, plus 0.25 U/L insulin, 10 μM cytosine-β-D-arabinofuranoside, 5% FCS, 0.05 mg/mL gentamycin [gibco®, Grand Island, NY, United States] in medium 199). Cardiomyocytes (17,500 cells/mL) were cultured on laminin (100 μg/mL) coated coverslips. Medium was exchanged 3–4 h after seeding cells, and cells were either measured after 48 h (control cells) or treated with adenovirus. Adenovirus (type 5) coding for GtACR1 was added immediately after the 3–4 h medium exchange (MOI75 for 48–72 h). Medium was renewed after 48–72 h of transduction immediately before cells were used for functional experiments.

### Patch-Clamp Recordings

Whole-cell patch-clamp measurements on single isolated cardiomyocytes or HEK 293T cells were performed at room temperature using an inverted DMI 4000B microscope (Leica Microsystems, Wetzlar, Germany), an Axopatch 200B amplifier and an Axon Digidata 1550A (Molecular Devices, San José, CA, United States). Activation light was delivered by a 525-nm LED (Luminus Devices PT-120-G, Sunnyvale, CA, United States) using a 530/20x filter and controlled *via* custom-built hardware (Essel Research and Development, Toronto, Canada). Light intensity in the object plane was determined with an optical power meter (PM100D, Thorlabs; Newton, NJ, United States) and the illuminated area was determined using a stage micrometer (*A* = 0.8 mm^2^). LED-input power was controlled via the custom-built LED control unit. If not specified otherwise, external (bath) and internal (pipette) standard solutions were used (Tables 3, 4).

**Table 3:** Composition of bath solutions used in whole-cell patch-clamp experiments.

**Components of external bath solution [mM]**	**Standard solution**	**Alkaline solution**	**Strongly reduced Na^**+**^ concentration**	**Moderately reduced Na^**+**^ concentration**	**Strongly reduced Cl^**−**^ concentration**	**Moderately reduced Cl^**−**^ concentration**	**Moderately increased K^**+**^ concentration**	**Strongly increased K^**+**^ concentration**
NaCl	140	140	1	12	-	60	140	140
Na-aspartate	-	-	-	-	140	80	-	-
Tetraethyl-ammoniumchloride	-	-	139	127	-	-	-	-
K-aspartate	-	-	-	-	-	-	6.6	21.6
KCl	5.4	5.4	5.4	5.4	5.4	5.4	5.4	5.4
CaCl_2_	1	1	1	1	1	1	1	1
MgCl_2_	2	2	2	2	2	2	2	2
Glucose	10	10	10	10	10	10	10	10
HEPES/TRIS	10/-	-/10	10/-	10/-	10/-	10/-	10/-	10/-
pH	7.4	9	7.4	7.4	7.4	7.4	7.4	7.4

**Table 4:** Composition of pipette solutions used in whole-cell patch-clamp experiments.

**Components of internal (pipette) solutions [mM]**	**Standard solution**	**Strongly reduced Cl^**−**^ concentration**	**Moderately reduced Cl^**−**^ concentration**
KCl	50	-	11
K-aspartate	80	130	119
MgCl_2_	2	2	2
Mg-ATP	3	3	3
EGTA	10	10	10
HEPES	10	10	10

*All solutions were adjusted to pH 7.2*.

#### Photoinhibition Protocol

In current-clamp mode AP were triggered at 0.25 Hz by current injection of 50% more than the threshold to elicit an AP using a current ramp from 0 pA within 10 ms. Light (525 nm for 64, 32, or 16 s at 4 mW·mm^−2^ or 0.4 mW·mm^−2^) was applied after 15 electrically-triggered AP, and at least 15 AP were recorded after light exposure. AP durations were determined at the 20th or 90th percentile of signal amplitude above resting potential, as earlier described (Wang et al., 2015). Depolarizations with peak amplitudes below 0 mV were marked as inhibited.

#### Light Titration

Maximal amplitude of inward currents was measured at −60 mV after light application for 1, 10, 100, 300 ms at light intensities of 0.1, 0.2, 0.4, 0.7, 1, 2, 4, 7, 10, and 11 mW·mm^−2^. The dissociation constant K_d_ was calculated by fitting the titration curves with a Hill equation.

#### Ion Selectivity Measurements

Maximal amplitudes of in- and outward currents were measured at different holding potentials (cardiomyocytes: −100 mV to +60 mV in steps of 20 mV, HEK cells: −60 mV to +60 mV in steps of 20 mV) during light exposure (525 nm, 300 ms, 4 mW·mm^−2^).

For titration of the Cl^−^ concentration, different solutions (strongly reduced external Cl^−^, moderately reduced external Cl^−^ and standard external) were used in combination with standard internal solution, and the reversal potential was determined from the IE curves. Different internal solutions (strongly reduced internal Cl^−^ and moderately reduced internal Cl^−^) were tested in combination with standard external solution (Table 2).

pH dependency experiments in standard external solution (pH = 7.4) were compared with external alkaline solution at pH 9.0 (Table 3).

For titration of Na^+^ concentration effects, three external solutions (strongly reduced and moderately reduced Na^+^ concentration, and standard external solution, Table 3) were used in combination with standard internal solution (Table 4). Similarly, extracellular K^+^ effects were assessed by exchanging the standard external solution by solutions with either strongly increased or moderately increased K^+^ concentration (Table 3).

The osmolality of all solutions was determined using a semimicro osmometer K-7400 (Knauer AG, Berlin, Germany) and adjusted to 300 ± 5 mOsm with glucose.

#### Peak Current Recovery

Cardiomyocytes were clamped at −60 mV and peak current amplitudes were recorded while optically stimulating for 300 ms at 11 mW·mm^2^. In dual-stimulation experiments peak recovery was probed by increasing dark intervals in-between stimuli from 1 to 20 s, in 1-s steps.

#### Optical Pacing

Cardiomyocyte membrane potential was recorded in current clamp mode at 0 pA. Myocytes were paced at 0.25 Hz with light pulses of 10 ms using either the threshold light intensity (*P*/*A*_thr_, minimal light intensity for 100% capture) or twice the threshold light intensity (*P*/*A*_2·*thr*_).

### Sarcomere Length Measurements

Cardiomyocyte contractions were followed by detecting changes in sarcomere length as previously described (Peyronnet et al., 2017). Cardiomyocytes were imaged with a MyoCam-S camera (IonOptix, Dublin, Ireland) on an inverted microscope (DM IRBE, Leica Microsystems, Wetzlar, Germany). Sarcomere length was calculated in real time by a Fast Fourier Transform of the power spectrum of the striation pattern (IonWizard, IonOptix). Cells were field stimulated at 14–15 V/0.25 Hz with a Myopacer (IonOptix). For the contraction inhibition protocol, a light pulse of 525 nm was applied for 64 s. For OP, light pulses of 10 ms were applied at 0.25 Hz. Resting sarcomere length, time to peak, time to 90% relaxation, fractional sarcomere shortening, maximum velocity of contraction and relaxation were analyzed as described before (Peyronnet et al., 2017).

### Confocal Microscopy of GtACR1-eGFP Expressing Cardiomyocytes

Transduced cardiomyocytes were washed two times with Dulbecco's Phosphate Buffered Saline (PBS). Ice-cold acetone was added, the samples were kept for 5 min at −20°C and washed again with PBS. Cells on coverslips were fixed with Mountant, Perma Fluor (Thermo Fisher Scientific) and flipped onto an object slide. The mounted samples were imaged with a confocal laser-scanning microscope (LSM 880; Carl Zeiss Microscopy, Oberkochen, Germany).

### Generation of cmlc2:GtACR1-eGFP Transgenic Zebrafish

All experimental procedures in zebrafish were approved by the Dalhousie University Committee for Laboratory Animals and followed the guidelines of the Canadian Council on Animal Care. Details of experimental protocols are reported following the Minimum Information about a Cardiac Electrophysiology Experiment (MICEE) reporting standard (Quinn et al., 2011).

A cmlc2:GtACR1-eGFP plasmid was generated using standard cloning and microbiology techniques combined with the multisite Gateway system for Tol2 transposon transgenesis (Rafferty and Quinn, 2018). The 5′ entry plasmid p5E-cmlc2 and destination plasmid pDestTol2pA2 were obtained from Dr. Ian Scott (University of Toronto, Toronto, Canada). The donor plasmid pME-TA and 3′ entry plasmid p3E-polyA were obtained from Dr. Jason Berman (Dalhousie University, Halifax, Canada). To generate the middle entry plasmid, the sequence of the L13_CMV_GtACR1-eGFP plasmid was confirmed (using primers CMV_Forward: 5′-CGCAAATGGGCGGTAGGCGTG-3′ and EGFP-C-REV: 5′-GTTCAGGGGGAGGTGTG-3′) and then GtACR1-eGFP was amplified out of the plasmid using polymerase chain reaction (PCR) with primers GtACR1_for: 5′-GCCACCATGAGCAGCATTAC-3′ and EGFP-stop_rev: 5′-TTTACTTGTACAGCTCGTCCAT-3′. The GtACR1-eGFP PCR product, purified using a Gel Extraction Kit (QIAEX II, Qiagen; Hilden, Germany) and with the addition of a polyA (pA) tail was ligated into pME-TA to generate the middle entry plasmid pME-GtACR1-eGFP-pA, which was then subjected to restriction digest and sequencing (using primers M13F(-21): 5′-TGTAAAACGACGGCCAGT-3′, GtACR1, and EGFP_stop_rev). An expression plasmid was then generated by ligating p5E-cmlc2, pME-GtACR1-eGFP-pA, and p3E-polyA into pDestTol2pA2. Restriction digest and sequencing (using primers T7: 5′-TAATACGACTCACTATAGGG-3′ and EGFP-stop_rev) confirmed the sequence of isolated expression plasmid DNA. The cmlc2-GtACR1-eGFP-pA-pDestTol2pA2 DNA was purified with a PCR Purification Kit (QIAquick, Qiagen) before microinjection of 22.7 ng/μL DNA and 16 ng/μL Tol2 transposase mRNA into one-cell stage Casper zebrafish embryos. Injected fish were screened for successful gene transduction by eGFP expression using a fluorescent microscope (MZ16F, Leica; Wetzlar, Germany).

### Zebrafish Isolated Heart Preparation

Hearts were isolated from 3-month post fertilization (mpf) zebrafish expressing GtACR1-eGFP as previously described (Stoyek et al., 2016, 2018; MacDonald et al., 2017). Fish were anesthetized in Tris-buffered (pH 7.4, BP152, Fisher Scientific; Hampton, United States) tricaine (1.5 mM MS-222) in room temperature tank water until opercular movements ceased and the animals lacked response to fin pinch with forceps. A midline incision was made through the ventral body wall and tissues of the ventral aorta, ventricle, atrium, and venous sinus were removed, pinned into a 15 mL dish lined with Sylgard (DC 170, Dow Corning; Midland, United States) containing Tyrode's solution (in mM: 142 NaCl, 4.7 KCl, 1 MgCl_2_, 1.8 CaCl_2_, 10 Glucose, 10 HEPES) of an osmolality of 300 ± 5 mOsm checked with an osmometer (Model 5004 μOsmette, Precision Systems; Natick, United States) and with pH titrated to 7.4 with NaOH. In addition, 10 μM (±)-blebbistatin (B592490, Toronto Research Chemicals; Toronto, Canada), a myosin inhibitor used for excitation-contraction uncoupling was added to the bath to eliminate contraction for stability of intracellular microelectrode recordings.

### Intracellular Microelectrode Recordings of Zebrafish Ventricular Myocyte Membrane Potential

Intracellular microelectrode recordings were performed as previously described (Smith et al., 2016). Microelectrodes made from borosilicate glass tubing (0.5 mm inner diameter, 1.0 mm outer diameter, with internal filament; type BF/100/50/10, Sutter Instruments; Novato, United States) were pulled on a Brown/Flaming micropipette puller (Model P97, Sutter Instruments) to tip diameters resulting in a 40–60 MΩ resistance when filled with 3 M KCl. Electrodes were coupled to the headstage of an amplifier (Model 1600 Neuroprobe Amplifier, A/M Systems; Everett, United States) operated in current clamp mode with an electrode holder (ESW-M10N, Warner Instruments; Hamden, United States). Electrodes were advanced with a mechanical manipulator (MX/4, Narishige; Tokyo, Japan) into the ventricular epicardium. Before cell penetration, the tip potential of the electrode was nulled using the bridge controls of the intracellular amplifier with the electrode tip in the bath. At the end of a recording, the microelectrode was withdrawn from the cell, the null potential was checked, and the previous data adjusted if necessary. Transmembrane potential was taken as the difference between the potential measured in the bath with a silver/silver-chloride lead and the intracellular potential. Successful impalement was signaled by a sudden step of the electrode potential to a negative value. Criteria for accepting a cell were AP with a stable resting membrane potential (*E*_R_) < −60 mV and an AP peak >0 mV that were maintained throughout the recording. Transmembrane potential was recorded at 10 kHz using a software-controlled data acquisition system (LabChart and PowerLab, ADInstruments; Sydney, Australia).

### Activation of GtACR1 in Zebrafish Isolated Hearts

Experiments were performed in zebrafish isolated hearts and intact zebrafish larvae at room temperature using an upright microscope (BX51WI, Olympus; Shinjuku, Japan). Light for activation of GtACR1 was delivered by a white LED (CFT-90-W, Luminus Devices; Sunnyvale, USA) through a 531/22 nm filter (FF02-531/22, Semrock; Rochester, USA) focused on the ventricle with a 10X (for isolated hearts) or 20X (for zebrafish larvae) water-immersion objective (UMPLFLN 10XW or XLUMPLFLN 20XW, Olympus). The microscope field stop was set at the smallest aperture to produce a 0.16 mm^2^ spot with an intensity of 1–5 mW/mm^2^. Light application was either sustained for 15 s or pulsed for 10 ms at a rate 2 × sinus heart rate (HR) in regions displaying high or no eGFP expression, checked with 466/40 nm excitation (FF01-466/40, Semrock), 495 nm dichroic (FF495-Di03, Semrock), and 525/50 nm (ET525/50m, Chroma Technology; Bellows Falls, United States) filters. OP was compared to EP at the same rate applied extracellularly by a pair of silver wire electrodes on either side of the heart coupled to a constant current stimulus isolation unit (PSIU6D, Grass Technologies; West Warwick, USA) driven by 10 ms rectangular pulses from a waveform generator (S44, Grass). Measurements of HR, *E*_R_, upstroke velocity (d*E*/d*t*_max_), AP amplitude (AP_Amp_), APD_50_ and APD_90_ were averaged over three consecutive heart beats before and during light stimulation. Light intensity was measured at the end of the experiment using a USB power meter (PM16-120, Thorlabs; Newton, United States).

### Confocal Microscopy of Ventricular cmlc2:GtACR1-eGFP Expression

GtACR1-eGFP expression in the ventricle was investigated in two subsets of zebrafish isolated hearts by confocal microscopy. The first subset included specimens from activation of GtACR1 in zebrafish isolated heart experiments. In this group immediately following the activation experiment isolated hearts were immersed in 2% methyl-cellulose on a depression slide, put under a cover slip, and GFP expression imaged. In the second subset, whole hearts were labeled with an antibody against GFP (1:100, PA1-980A, Thermo Fisher Scientific; Waltham, United States) to enhance the signal associated with the eGFP tagged to ACR1. Tissues were fixed overnight in 2% paraformaldehyde (RT-15710, Electron Microscopy Sciences; Hatfield, United States) with 1% dimethyl sulfoxide in phosphate-buffered saline (PBS, in mM): 140 NaCl, 50 Na_2_HPO_4_, with pH titrated to 7.2 with NaOH. Fixed tissues were rinsed in PBS, and then incubated with primary GFP-antibody diluted in a solution containing 0.1% Triton X-100 in PBS (PBS-T) for 36 h at room temperature with gentle agitation. Tissues were then rinsed and transferred to a solution of PBS-T containing anti-rabbit AlexaFluor 488 (1:200, A-21206, Life Technologies; Carlsbad, United States) for 24 h at room temperature with gentle agitation. Final rinsing was done with PBS and specimens were placed in Scale CUBIC-1 clearing solution (Susaki et al., 2014) overnight at room temperature with gentle agitation. Tissues were mounted on glass slides in CUBIC-1 for confocal microscopy.

In both subsets, processed specimens were examined as whole-mounts using an LSM 510 confocal microscope (Carl Zeiss Microscopy) with a 10 × , 0.45 NA objective (Plan-Apochromat SF25, Carl Zeiss) or a 25 × , 0.80 NA objective (LCI Plan-Neofluar, Carl Zeiss). Preparations were epi-illuminated with a 488 nm argon laser reflected by a 488 nm dichroic mirror (HFT 488; Carl Zeiss). Emitted fluorescence was collected using a 500–530 nm band-pass filter (Carl Zeiss AG). Confocal image Z-stacks ranging from 20 to 50 μm in depth, including a region of 2–5 μm above and below the region of interest (to ensure that all structures were captured, while limiting issues of light scattering) were acquired and processed using Zen2009 software (Carl Zeiss) from regions surrounding immunoreactive tissues.

### Data Analysis

Data was analyzed with pClamp 11, IonWizard, OriginPro, and with custom routines in Matlab. For unpaired datasets the non-parametric Mann-Whitney-test was applied. The Wilcoxon-test and two-tailed paired Student's *t*-test were used to pair-wise compare two datasets. For more than two datasets ANOVA one-way repeated (*post*-*hoc* test: Tukey or Dunnett) was used. A *p* < 0.05 was taken to indicate a significant difference between means.

## Data and Material Availability

The raw data supporting the conclusions of this manuscript will be made available by the authors, without undue reservation, to any qualified researcher. DNA plasmids and established zebrafish lines will be provided upon request.

## Author Contributions

RK, TQ, PK and FS-W designed the experiments. RK, JB, SR, RM, CZ-J, SP, MS, FS, TQ and FS-W performed the experiments and analyzed the data. RK, CZ-J, FS, MS, TQ, PK and FS-W wrote the manuscript and designed the figures. All authors approved the final manuscript.

### Conflict of Interest Statement

The authors declare that the research was conducted in the absence of any commercial or financial relationships that could be construed as a potential conflict of interest. The handling editor and reviewer CH declared their involvement as co-editors in the Research Topic, and confirm the absence of any other collaboration.

## Supplementary Material

Supplementary Figure 1 **(A)** *IE* relationship of GtACR1 currents at various internal chloride concentrations in HEK 293T cells (*n* = 7–12). **(B)** *IE* relationship of GtACR1 currents at various external K^+^ concentrations in HEK 293T cells (*n* = 7–12). **(C)** *IE* relationship of GtACR1 currents at various internal chloride concentrations in cardiomyocytes (*n* = 7–17, *N* = 4). **(D)** *IE* relationship recorded at pH 7.4 and pH 9.0 (*n* = 17–20, *N* = 4). **(E)** Change of resting membrane potential during electrical and optical pacing of cardiomyocytes. Mann-Whitney test: *p* > 0.05 (n.s.), Wilcoxon test: *p* < 0.01 (^**^, *n* = 10, *N* = 2). **(F)** Change of resting membrane potential *E*_*R*_ during optical pacing (OP) at threshold light intensity and two times threshold intensity. Wilcoxon test: *p* < 0.05 (^*^). **(G)** Resting membrane potential and **(H)** APD_90_ in control wild-type cells during optical inhibition protocol (*n* = 10, *N* = 1). AP are not inhibited by light. ANOVA one-way repeated Dunnett test: n.s. **(I-N)** Quantification of key parameters of cellular contractions in sarcomere length measurements (*n* = 12–17, *N* = 3). **(I)** Resting sarcomere length (RSL, *p* ≤ 0.05 [^*^]), **(J)** time to peak, a parameter for the active shortening, **(K)** time from peak to 90% relaxation, **(L)** fractional sarcomere shortening, the percentage of the peak amplitude in relation to the RSL, **(M)** maximum velocity of contraction and **(N)** relaxation were analyzed for 6 contractions before and after light exposure. Wilcoxon was used to compare paired data, Mann-Whitney for comparison of control cells vs. GtACR1-expressing cells before light.

Supplementary Figure 2 Representative traces of cardiomyocyte membrane potential during electrical pacing (EP) at 0.25 Hz (current injections of 0.6 nA for 10 ms). AP generation is inhibited by green light application for 64 s **(A)**, 32 s **(B)** and 16 s **(C)**.

Supplementary Figure 3 Confocal microscopy optical slice through the ventricular mid-wall of an immunohistochemically labeled zebrafish isolated heart. Anti-GFP reveals heterogeneous cellular expression of GtACR1-eGFP (indicated by areas of bright green; the duller green represents tissue autofluorescence). The region of high intensity at the apex illustrates epicardial expression (as the optical slice goes through the epicardium in this region), while the bright band in the center of the image represents high expression in trabecular muscle.

Supplementary Figure 4 Comparison of OP and EP at the same rate shows no difference in the resulting AP morphology of a ventricular cell inside an isolated heart of zebrafish 3 mpf.

Supplementary Movie 1 Inhibition of ventricular contraction by exposure of the entire ventricle to 1.7 mW·mm^−2^ light in an intact zebrafish larva 21 days post fertilization.
