# Supplementary figures and images for: Cardiac Electrophysiological Effects of Light-Activated Chloride Channels
Source: Front Physiol. 2018 Dec 17;9:1806. doi: 10.3389/fphys.2018.01806 (PMC6304430; doi:10.3389/fphys.2018.01806)

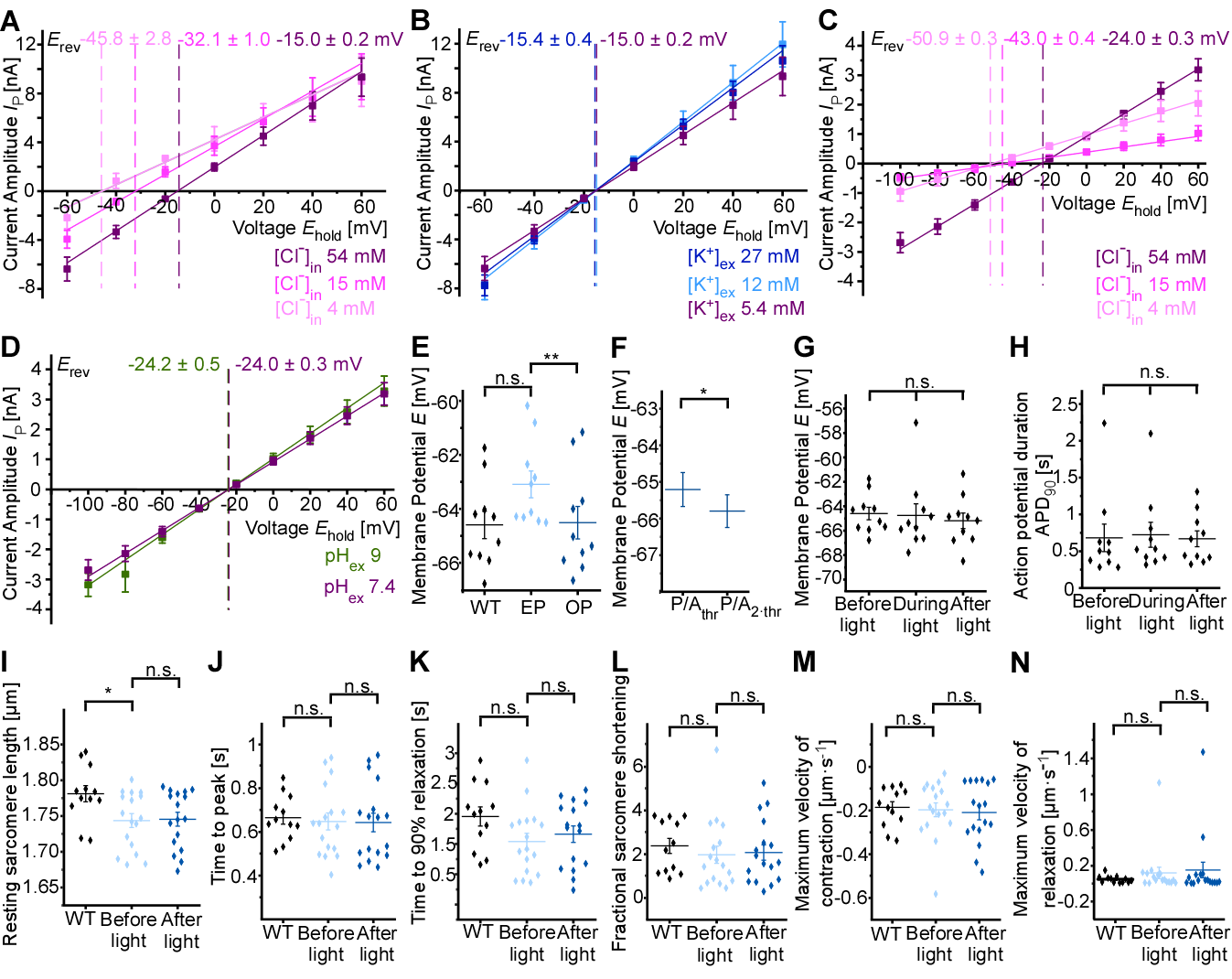

Supplement: Supplementary Figure 1 — (A) IE relationship of GtACR1 currents at various internal chloride concentrations in HEK 293T cells (n = 7–12). (B) IE relationship of GtACR1 currents at various external K+ concentrations in HEK 293T cells (n = 7–12). (C) IE relationship of GtACR1 currents at various internal chloride concentrations in cardiomyocytes (n = 7–17, N = 4). (D) IE relationship recorded at pH 7.4 and pH 9.0 (n = 17–20, N = 4). (E) Change of resting membrane potential during electrical and optical pacing of cardiomyocytes. Mann-Whitney test: p > 0.05 (n.s.), Wilcoxon test: p < 0.01 (**, n = 10, N = 2). (F) Change of resting membrane potential ER during optical pacing (OP) at threshold light intensity and two times threshold intensity. Wilcoxon test: p < 0.05 (*). (G) Resting membrane potential and (H) APD90 in control wild-type cells during optical inhibition protocol (n = 10, N = 1). AP are not inhibited by light. ANOVA one-way repeated Dunnett test: n.s. (I-N) Quantification of key parameters of cellular contractions in sarcomere length measurements (n = 12–17, N = 3). (I) Resting sarcomere length (RSL, p ≤ 0.05 [*]), (J) time to peak, a parameter for the active shortening, (K) time from peak to 90% relaxation, (L) fractional sarcomere shortening, the percentage of the peak amplitude in relation to the RSL, (M) maximum velocity of contraction and (N) relaxation were analyzed for 6 contractions before and after light exposure. Wilcoxon was used to compare paired data, Mann-Whitney for comparison of control cells vs. GtACR1-expressing cells before light. [file Image_1.TIF]

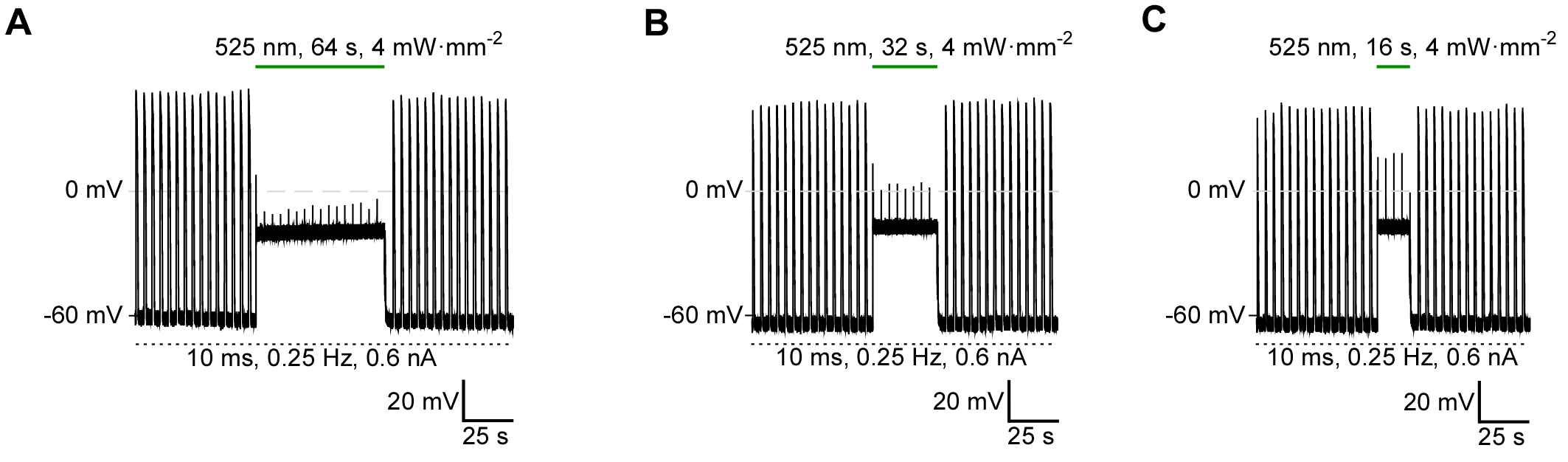

Supplement: Supplementary Figure 2 — Representative traces of cardiomyocyte membrane potential during electrical pacing (EP) at 0.25 Hz (current injections of 0.6 nA for 10 ms). AP generation is inhibited by green light application for 64 s (A), 32 s (B) and 16 s (C). [file Image_2.TIF]

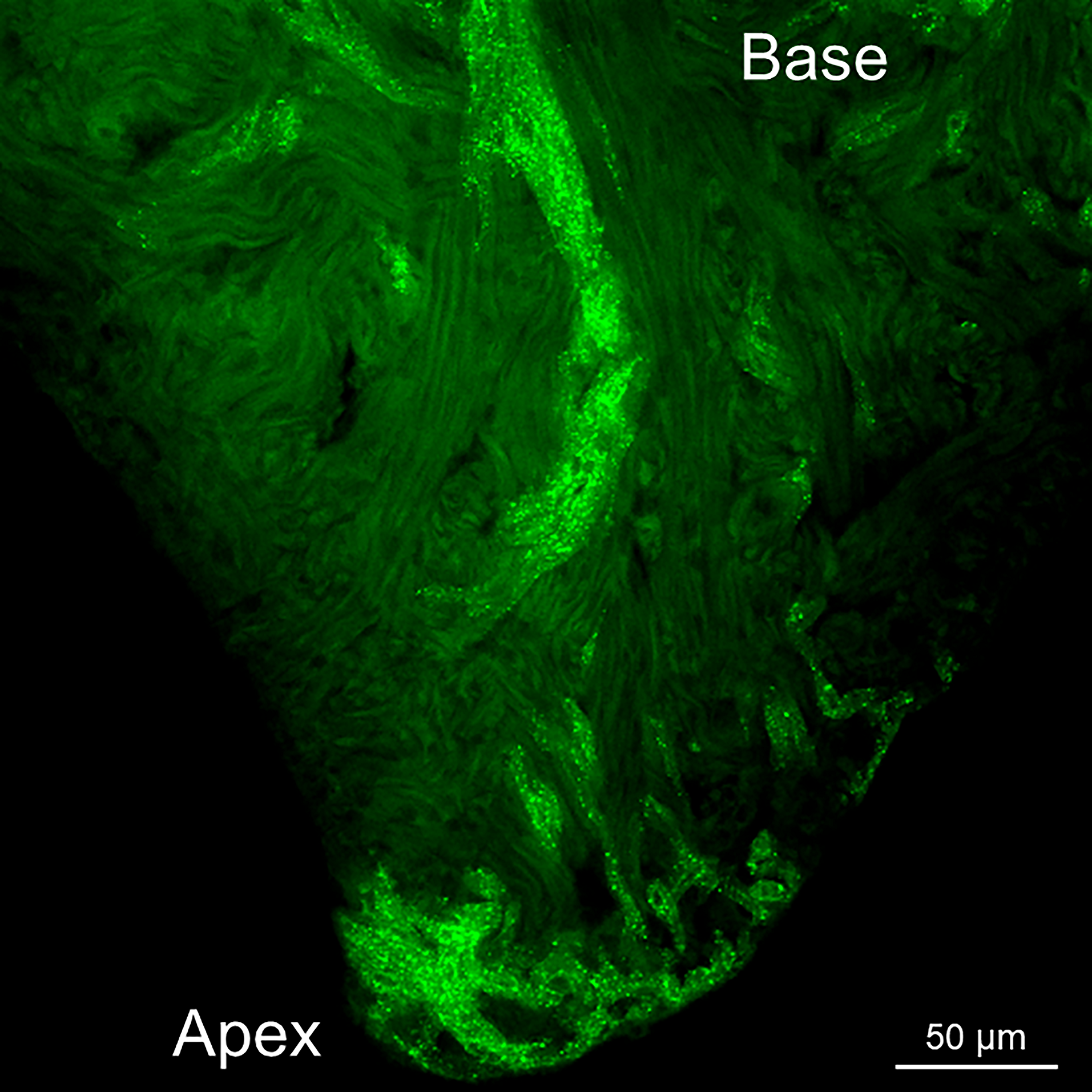

Supplement: Supplementary Figure 3 — Confocal microscopy optical slice through the ventricular mid-wall of an immunohistochemically labeled zebrafish isolated heart. Anti-GFP reveals heterogeneous cellular expression of GtACR1-eGFP (indicated by areas of bright green; the duller green represents tissue autofluorescence). The region of high intensity at the apex illustrates epicardial expression (as the optical slice goes through the epicardium in this region), while the bright band in the center of the image represents high expression in trabecular muscle. [file Image_3.TIF]

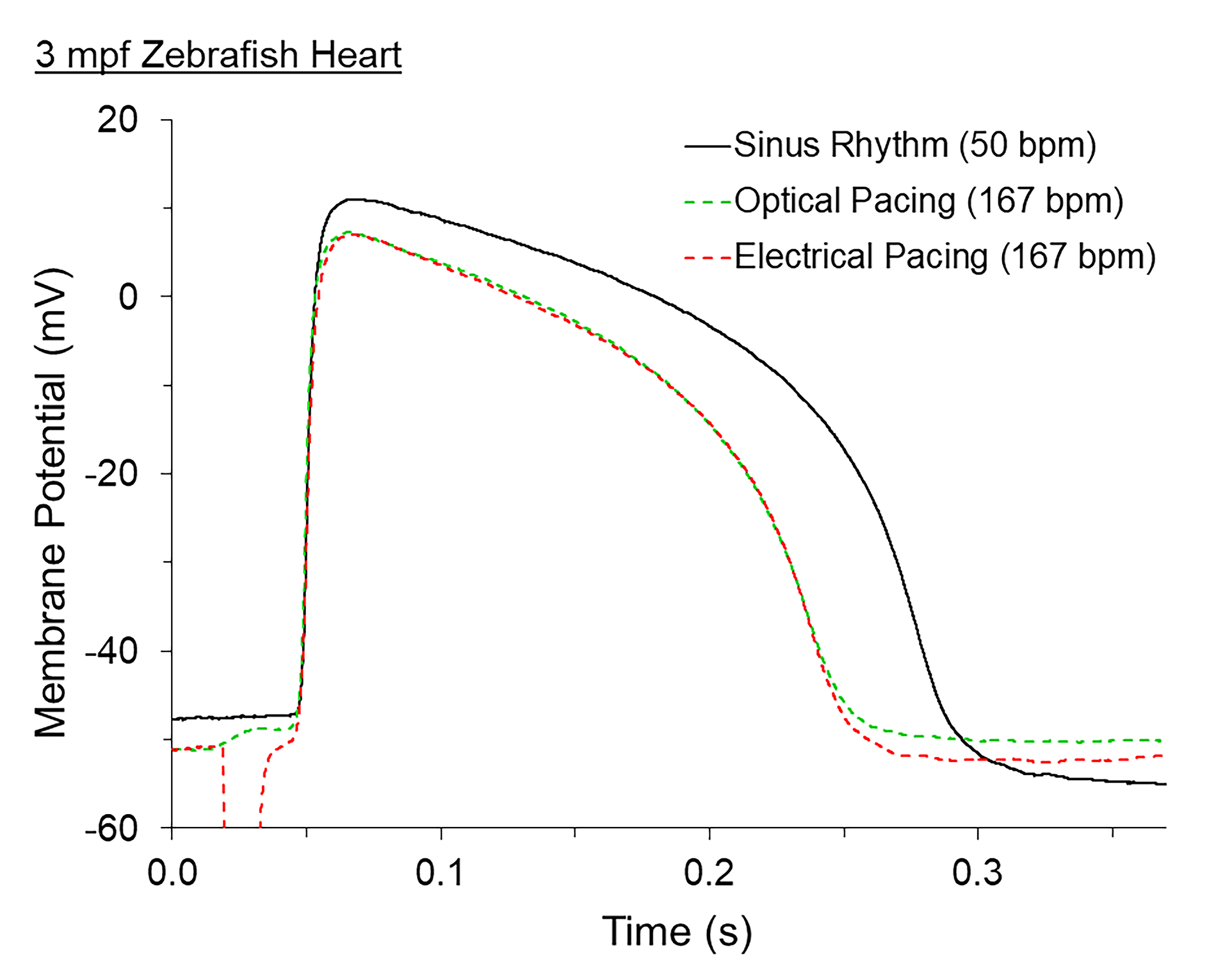

Supplement: Supplementary Figure 4 — Comparison of OP and EP at the same rate shows no difference in the resulting AP morphology of a ventricular cell inside an isolated heart of zebrafish 3 mpf. [file Image_4.TIF]
